# Epigenetic regulation of GABAergic differentiation in the developing brain

**DOI:** 10.3389/fncel.2022.988732

**Published:** 2022-09-23

**Authors:** Juanmei Gao, Yuhao Luo, Yufang Lu, Xiaohua Wu, Peiyao Chen, Xinyu Zhang, Lu Han, Mengsheng Qiu, Wanhua Shen

**Affiliations:** ^1^College of Life Sciences, Zhejiang University, Hangzhou, China; ^2^Zhejiang Key Laboratory of Organ Development and Regeneration, College of Life and Environmental Sciences, Hangzhou Normal University, Hangzhou, China

**Keywords:** histone (de)acetylation, GABA, differentiation, *Xenopus*, optic tectum, VPA

## Abstract

In the vertebrate brain, GABAergic cell development and neurotransmission are important for the establishment of neural circuits. Various intrinsic and extrinsic factors have been identified to affect GABAergic neurogenesis. However, little is known about the epigenetic control of GABAergic differentiation in the developing brain. Here, we report that the number of GABAergic neurons dynamically changes during the early tectal development in the *Xenopus* brain. The percentage of GABAergic neurons is relatively unchanged during the early stages from stage 40 to 46 but significantly decreased from stage 46 to 48 tadpoles. Interestingly, the histone acetylation of H3K9 is developmentally decreased from stage 42 to 48 (about 3.5 days). Chronic application of valproate acid (VPA), a broad-spectrum histone deacetylase (HDAC) inhibitor, at stage 46 for 48 h increases the acetylation of H3K9 and the number of GABAergic cells in the optic tectum. VPA treatment also reduces apoptotic cells. Electrophysiological recordings show that a VPA induces an increase in the frequency of mIPSCs and no changes in the amplitude. Behavioral studies reveal that VPA decreases swimming activity and visually guided avoidance behavior. These findings extend our understanding of histone modification in the GABAergic differentiation and neurotransmission during early brain development.

## Introduction

In the central nervous system, glutamate and γ-amino butyric acid (GABA) are the two most abundant neurotransmitters in the vertebrate brain. GABAergic neurons and GABAergic signaling serve essential roles in the establishment and refinement of neural circuits during brain development, including their feedforward and feedback inhibition in preventing hyperexcitability and epileptiform activity (Ben-Ari, 2002; Hensch and Fagiolini, 2005; Akerman and Cline, 2007; Shen et al., 2009). Previous studies obtained from *in vivo Xenopus* tectum have demonstrated that both GABAergic cells and GABAergic neurotransmission are required to control the balance of excitation to inhibition, which in turn shapes the maturation of neural circuits during early brain development (Zhang et al., 1998; Wehr and Zador, 2003; Tao and Poo, 2005; Akerman and Cline, 2006; Shen et al., 2009, 2011; Richards et al., 2010; He et al., 2016). Although the anatomical distribution of GABAergic neurons is well documented in the *Xenopus* brain (Li et al., 2006; Miraucourt et al., 2012), less is understood about the underlying mechanisms of epigenetic regulation of GABAergic neurogenesis and differentiation in the developing brain.

Histone acetylation, a most characterized histone modification, is widely accepted to regulate neural proliferation and differentiation by changing transcriptional activity (Graff and Tsai, 2013a; Tao et al., 2015; Gao et al., 2016b). Valproic acid (VPA), a class I/II histone deacetylase (HDAC) inhibitor, is a primary drug used to treat epilepsy and bipolar mood disorder (Graff and Tsai, 2013b). The role of HDACs in neuronal proliferation and differentiation is still obscure, according to various studies in different experimental conditions. Several previous studies have shown that VPA or trichostatin A (TSA) treatment increases neurogenesis and neurotrophic action through the induction of neurogenic transcription factors (Hao et al., 2004; Laeng et al., 2004; Balasubramaniyan et al., 2006; Siebzehnrubl et al., 2007; Yu et al., 2009; Zhang et al., 2011). However, other studies have reported that the application of VPA decreases the number of GAD67-positive neurons or GABAergic synapses with impaired miniature IPSCs in cultured cortical neurons or brain slices (Fukuchi et al., 2009; Banerjee et al., 2013; Kumamaru et al., 2014; Iijima et al., 2016). In light of this controversy, it is essential to examine the effect of VPA on GABAergic neurogenesis and GABAergic neurotransmission in the early developing brain *in vivo*.

The *Xenopus* has been considered a reliable *in vivo* animal model system, which is widely used to study brain development, neural plasticity, and neurological diseases (Ruthazer and Aizenman, 2010; Pratt and Khakhalin, 2013; Igarashi et al., 2015; Paredes et al., 2015; Pratt et al., 2016; Thompson and Cline, 2016; Cioni et al., 2018; Gao and Shen, 2021). Here, we investigated the epigenetic control of GABAergic differentiation and neurotransmission in the early developing *Xenopus* brain. We found that the number of GABAergic neurons is dynamically decreased, accompanied by downregulation of H3K9 histone acetylation during the early optic tectum development from stages 46 to 48. Interestingly, VPA treatment at stage 46 for 48 h increases the number of GABAergic neurons and GABAergic neurotransmission. Our findings raise the possibility that histone acetylation controls the specification of GABAergic neurons and their neurotransmission *in vivo*.

## Materials and methods

### Animals

All animal procedures were performed according to the requirements of the “Regulation for the Use of Experimental Animals in Zhejiang Province.” This study has been approved by the local ethics committee of Hangzhou Normal University. Adult male and female *Xenopus laevis* were injected with human chorionic gonadotropin (HCG) to produce tadpoles, which were raised on a 12 h dark/12 h light cycle in Steinberg’s solution [(in mM): 10 HEPES, 58 NaCl, 0.67 KCl, 0.34 Ca(NO_3_)_2_, 0.83 MgSO_4_, pH 7.4] within a 20°C incubator. For experimental manipulations, tadpoles were anesthetized in 0.02% MS-222 (3-aminobenzoic acid ethyl ester methanesulfonate, Sigma Aldrich). The stages of tadpoles were determined according to the anatomic characterization (Nieuwkoop and Faber, 1994).

### Immunohistochemistry and image analysis

Tadpoles were anesthetized and fixed in 4% paraformaldehyde (PFA, pH 7.4) at 4°C overnight. Tadpoles were washed with phosphate buffer (PB, 0.1 M, pH 7.4) and immersed in sucrose (30%) overnight for dehydration. Animals were cut into 20 μm cryostat sections with a microtome (Microm, HM550 VP) after being embedded in optimal cutting temperature (OCT) media. Sections were rinsed with PB for 2 × 20 min, permeabilized with 0.3% Triton X-100 in PB for 4 × 10 min, and blocked with goat serum for 30 min before incubating with primary antibodies at 4°C overnight. For primary antibodies, we used the antibodies of anti-GABA (1:1000, Rabbit, Sigma, A2052), anti-CaMKIIα (1:100, Mouse, Cell signaling technology, 50049), and anti-HuD (1:100, Mouse, Santacruz, sc-48421). Sections were rinsed with PB, incubated with secondary antibody (FITC, Rhod, or Alexa 647) for 1 h at room temperature, and then counterstained with DAPI. The first distinct tectal section with evident GABA-positive stainings was identified starting from the dorsal tectum. The 5 consecutive sections were selected in a whole optic tectum for further analysis. All sections were prepared, imaged, and analyzed in parallel across samples. Immunopositive cells were collected using a confocal microscope (LSM710, Zeiss, Germany) and counted by iMaris image processing software (Bitplane AG, Zurich) (Gao et al., 2016a,b).

### Western blot

Animals were anesthetized and the tecta were dissected. The optical tecta (approximately 30–50 brains for each group) were homogenized in radioimmune precipitation assay (RIPA) buffer with a protease inhibitor cocktail (Sigma Aldrich) and phenylmethylsulfonyl fluoride (PMSF, Solarbio) at 4°C. Protein concentrations were measured by BCA assay using a Nanodrop (Thermo Scientific, 2000c). Protein homogenates were separated by SDS-PAGE (Bio-Rad). PVDF membranes were blocked in 4% non-fat milk for 1 h with TBS buffer containing 0.1% Tween-20 (Sigma Aldrich) (TBST) and incubated with primary antibodies overnight at 4°C. Antibodies were diluted in 1% non-fat milk. We used the following antibodies: anti-acetylation H3K9 (1:2000, Rabbit, Abcam, ab10812), anti-GAD67 (1:20000, Rabbit, Sigma, G5163), anti-VGAT (1:2000, Rabbit, ABclonal, A3129), and anti-GAPDH (1:10000, Rabbit, Millipore, GR68497-2). Blots were rinsed with TBST and incubated with the following horseradish peroxidase (HRP)-conjugated secondary antibodies for 1 h at room temperature: goat anti-rabbit IgG (1:2000, CWbiotech, CW0103) and goat anti-mouse IgG (1:2000, CWbiotech, CW0102). Bands were visualized using ECL chemiluminescent (1:1, Pierce).

### Electrophysiology

Tadpole preparation for whole-cell recording was performed as described previously (Luo et al., 2021). All recordings were performed at room temperature (20–22°C). The optic tecta were dissected out and placed in a recording chamber. A suction electrode removed the superficial cells. Optic tecta were perfused with an external solution containing (in mM: 115 NaCl, 2 KCl, 3 CaCl_2_, 1.5 MgCl_2_, 5 HEPES, 10 glucose, 0.01 glycine and 100 nM TTX, pH 7.2, osmolality 255 mOsm). Miniature inhibitory postsynaptic currents (mIPSCs) were recorded by holding the membrane potential at 0 mV with an intracellular solution containing (in mM: 110 K-gluconate, 8 KCl, 5 NaCl, 1.5 MgCl_2_, 20 HEPES, 0.5 EGTA, 2 ATP, and 0.3 GTP). Recording micropipettes were pulled from borosilicate glass capillaries and had resistances of 7–9 MΩ. The liquid junction potential was adjusted during recording. Signals were filtered at 2 kHz with a MultiClamp 700B amplifier (Molecular Devices, Palo Alto, CA, United States). Data were sampled at 10 kHz and analyzed using MiniAnalysis (Synaptosoft).

### Behavior

All experiments were performed at stage 48 tadpoles. For free swimming behavioral test, tadpoles were placed in 24-well Falcon culture dishes for 60 s. Each well contained 2 mL of Steinberg’s solution and one tadpole. The swimming trajectory was monitored and analyzed for locomotion analysis by the Danio Vision system (Danio Vision, Noldus).

The visual avoidance behavior was measured with custom-made equipment according to previous studies (Shen et al., 2011). Single tadpoles were placed in a chamber (9 × 8 × 3 cm) filled with 1.5 cm Steinberg’s rearing solution. Visual stimuli for moving spots were presented on the bottom of the chamber with a back-projection screen using a microprojector (3 M, MPro110). Infrared LEDs illuminated tadpoles, and the videos were recorded with a digital camera (Full HD 1080P, 15 frames/s). Visual stimuli were generated and presented by MATLAB 2012a (The MathWorks, Psychophysics Toolbox toolbox). Randomly positioned moving spots of 4 mm diameter were presented for 60 s. Visual avoidance behavior was scored as a change in swim trajectory with the first ten encounters of the tadpoles and moving spots (plotted as avoidance index).

### Apoptosis assay

Cell apoptosis was assayed by a TUNEL (*in situ* terminal deoxynucleotidyl transferase deoxyuridyl triphosphate nick-end labeling) method (Beyotime, C1089). The tadpoles were fixed in 4% PFA overnight at 4°C. Cryosections of full optic tectum were cut from the OCT embedded brains. Slices were washed 3 times with PB (0.1 M) every 10 min and treated with 0.3% TritonX-100 in PBS for 30 min. After rinsing in PBS, the sections were incubated with a TUNEL reaction mixture (terminal deoxynucleotidyl transferase plus nucleotide mixture in reaction buffer) in the dark for 60 min at 37°C. Sections were rinsed with PBS, counterstained with DAPI and photographed using confocal microscopy (LSM710, Zeiss, Germany). All apoptotic cells were counted from 5 representative brain sections by iMaris software.

### Drugs and treatment

We incubated tadpoles with VPA (1 mM, Sigma-Aldrich, P4543), a broad class I/II HDACs inhibitor, to block histone deacetylase activity. If not stated otherwise, tadpoles were reared in Steinberg’s solution for 48 h.

### Statistics

Shapiro–Wilk test was used to carry out the normality test. Paired data were tested with Student’s *t*-test. Multiple group data were tested with ANOVA followed by *post-hoc* Tukey’s test unless noted. Data are represented as the mean ± SEM. Experiments and analysis were performed blind to the experimental conditions unless noted.

## Results

### The number of GABAergic neurons is developmentally regulated in the developing brain

**FIGURE 1:**
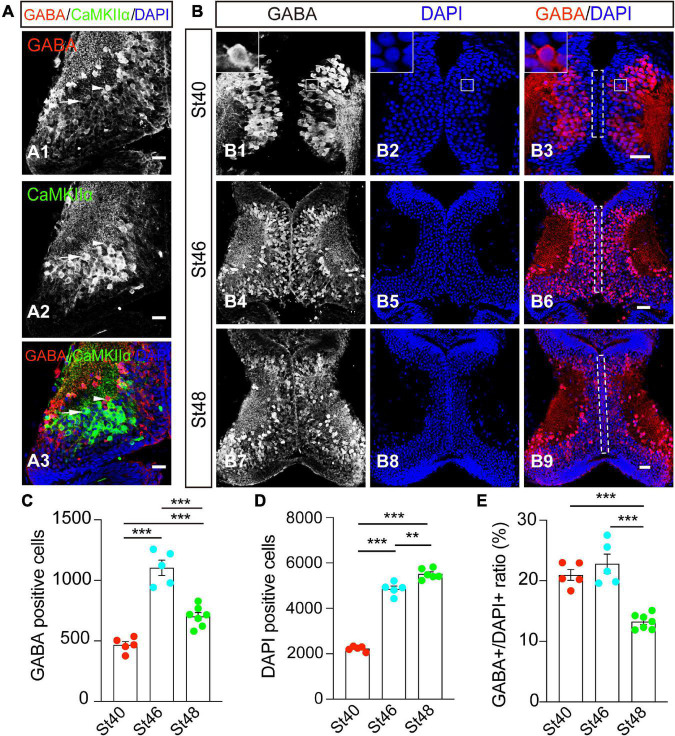
GABA^+^ cells are developmentally downregulated from stages 46 to 48 in the optic tectum. **(A)** The optic tectum was immunostained with anti-GABA and anti-CaMKIIα antibodies at stage 48. Arrowheads indicate the GABA^+^ and CaMKIIα^–^ cells. Arrows indicate the GABA^–^ and CaMKIIα^+^ cells. Scale bar = 50 μm. **(B)** The optic tectum was immunostained with an anti-GABA antibody at stages (St) 40 (B1–3), 46 (B4–6), and 48 (B7–9), respectively. The white line indicates the zoomed-in images that GABA is expressed in the cytoplasm (up-left corner, B1–3). The dotted line indicated the ventricular cell layer (B3,B6,B9). Scale bar = 50 μm. **(C)** The data shows the total number of GABA^+^ cells in the optic tectum. **(D)** The summary graph shows the total number of DAPI^+^ cells. **(E)** The summary graph shows the ratio of GABA^+^ cells to DAPI^+^ cells. (GABA: St40, 468 ± 27.0, *N* = 5, St46, 1103.8 ± 62.9, *N* = 5, St48, 723 ± 28.7, *N* = 6; DAPI: St40, 2227.4 ± 48.9, *N* = 5, St46, 4859 ± 125.5, *N* = 5, St48, 5515 ± 91.9, *N* = 7; **p < 0.01, ***p < 0.001). *N* represents the number of tadpoles.

γ-amino butyric acid is a marker for GABAergic inhibitory neurons, whereas the alpha isoform of the calcium/calmodulin-dependent protein kinase II (CaMKIIα), is a marker of mature excitatory neurons. To first characterize the cell identities in the developing optic tectum, we performed whole-brain immunostaining of an optic tectum using anti-GABA and anti-CaMKIIα antibodies (Figure 1A). At stage 48, the cryostat of the coronal section showed that GABA-positive (GABA^+^) cells were widely distributed within the optic tectum (Figure 1A1). Few GABA^+^ cells scattered in the tectal neuropil are also CaMKIIα-negative (Figure 1A3). The GABA-positive cells were not overlapping with CaMKIIα-positive cells, allowing us to identify two discrete subpopulations of GABAergic and glutamatergic neurons in the developing optic tectum (Figure 1A).

To measure the developmental changes in GABAergic neurons in the optic tectum, we counted GABAergic (GABA^+^) cells and total tectal cells (DAPI^+^) from the tectum at stages 40, 46, and 48 in 5 representative slices (Figure 1B) (see the section “methods”). The cells along the ventricular layer are brain lipid-binding protein (BLBP)-positive progenitor cells (Tao et al., 2015; Gao et al., 2016b). The majority of GABA^+^ cells are located within the cell body layer at stage 40 (Figure 1B1). No significant GABA staining was found in the ventricular layer (Figures 1B3,B6,B9). To compare the developmental dynamic of GABAergic cells, we counted the total GABA^+^ neurons from the entire tectum. We found that the number of GABAergic neurons developmentally increased from stage 40 to stage 46 but started to decrease from stage 46 to stage 48 (Figure 1C). The DAPI-positive tectal cells gradually increased during the tested stages from 40 to 48 (Figure 1D), indicating that the tectal growth was developmentally expanded in our tadpole rear conditions. The percentage of GABA^+^ cells was maintained from stage 40 (∼21%) to stage 46 (∼23%), but markedly decreased to 13% at stage 48 (Figure 1E). As a result, we reasoned that the dynamic changes of GABAergic neurons in the developmental optic tectum might allow us to investigate the underlying mechanism that controls the number of GABAergic inhibitory cells.

### Histone acetylation of H3K9 is developmentally downregulated in the optic tectum

**FIGURE 2:**
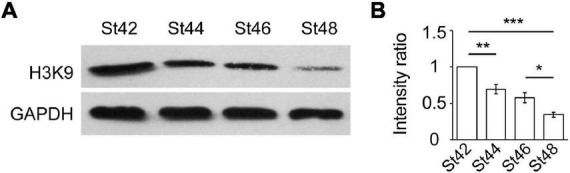
Histone acetylation of H3K9 is developmentally decreased from stage 42 to 48 optic tectum. **(A)** Western blot analysis of H3K9 protein expression using an anti-H3K9ac antibody at St42, St44, St46, and St48. **(B)** The data shows that the relative intensity of H3K9 at St48 was significantly decreased compared to St42 and St46 tadpoles (*N* = 3, *p < 0.05, **p < 0.01, ***p < 0.001). *N* represents the number of independent experiments.

Our previous studies have demonstrated that histone acetylation regulates neural proliferation and differentiation in the developing optic tectum (Tao et al., 2015; Gao et al., 2016b; Ruan et al., 2017). To determine whether histone acetylation is involved in the regulation of GABAergic cells during the early tectal development, the optic tecta were dissected, homogenized, and immunoblotted using an antibody against acetylation H3K9 (H3K9ac) at stages 42, 44, 46, and 48 tadpoles (Figure 2A). We observed that H3K9 acetylation gradually decreased following the tectal development from stage 42 to stage 48 tadpoles (Figure 2B). The acetylation level was lower at stage 48 compared to stages 42 and 46, suggesting that higher histone acetylation is required for early brain development. Combined with the decrease of GABA^+^ cells from stages 44 to 48, the changes in histone modification allow us to study epigenetic regulation of GABAergic differentiation in the developing brain.

### Valproate acid increases GABAergic differentiation and enhances histone acetylation

**FIGURE 3:**
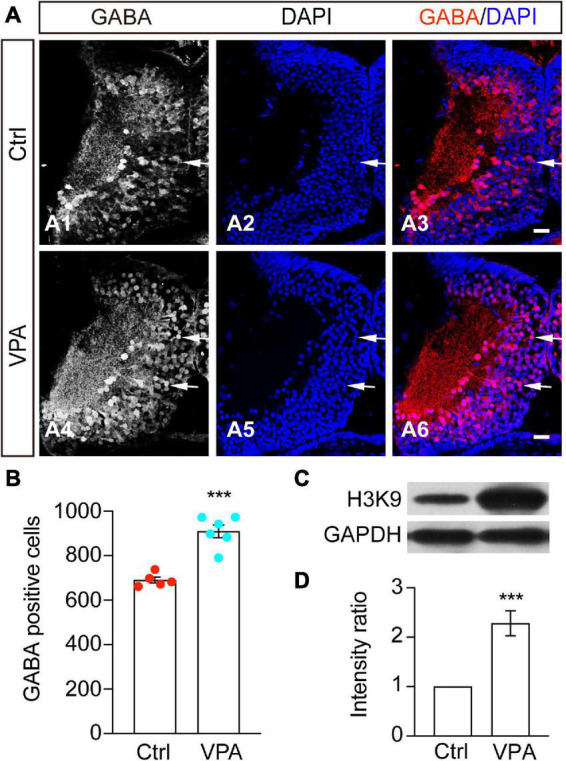
The number of GABA^+^ cells was increased by VPA treatment. **(A)** Representative images showing that the control (A1–3) and VPA-treated (A4–6) brains were immunostained with an anti-GABA antibody at stage 48. Arrows indicate the GABA^+^ cells. Scale bar = 50 μm. **(B)** The data shows that the GABA^+^ cells were significantly increased by VPA treatment. GABA: Ctrl, 690.2 ± 13.4, *N* = 5; VPA, 909.7 ± 28.3, *N* = 6). *N* represents the number of tadpoles. **(C,D)** Western blot of H3K9ac and summary of data showing that the relative intensity of H3K9 to GAPDH was significantly increased by VPA treatment. *N* = 12. ***p < 0.001. *N* represents the number of independent experiments.

Histone deacetylase inhibitors, such as VPA or TSA, can increase transcriptional activity by altering histone acetylation levels (Tao et al., 2015; Gao et al., 2016b). To test whether histone modification affects GABAergic differentiation, we exposed tadpoles to VPA (1 mM), a class I/II HDAC inhibitor, at stage 46 for 48 h. We first performed immunostaining analysis and observed that the number of GABA^+^ cells was markedly increased by VPA incubation (Figures 3A,B), indicating that VPA treatment affects GABAergic differentiation in the developing optic tectum. To test whether histone modifications mediate the VPA-induced increase of GABAergic neurons, we performed a Western blot analysis using an anti-H3K9ac antibody. We observed that VPA treatment significantly enhanced the acetylation levels of H3K9 (Figures 3C,D). These data suggest that the increase of histone acetylation is involved in the enhancement of GABAergic differentiation.

### Valproate acid-induced GABAergic cells are HuD-positive neurons

**FIGURE 4:**
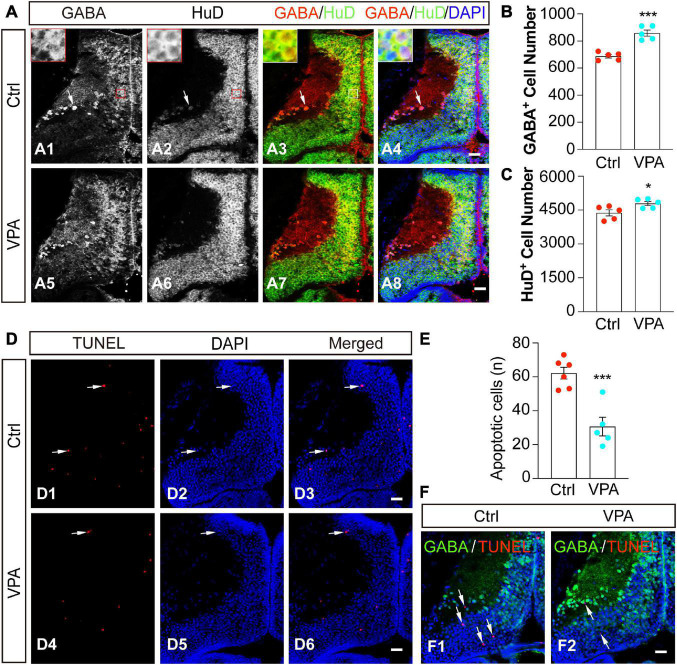
Valproate acid-induced GABA^+^ cells are HuD^+^ differentiated neurons. **(A)** Control (A1–4) and VPA-reared (A5–8) optic tecta were immunostained with anti-GABA and anti-HuD antibodies at stage 48. Red/white lines indicate zoomed-in images showing that GABA and HuD are overlapped in the cell body layer. Arrows indicate that GABA^+^ and HuD^+^ cells are distributed in the neuropil. Scale bar = 50 μm. **(B)** The data shows that the number of GABA^+^ cells was significantly increased in VPA-raised tadpoles compared to Ctrl. (Ctrl, 688.2 ± 13.3, *N* = 5; VPA, 858.8 ± 22.3, *N* = 5; ***p < 0.001). **(C)** The data shows that the number of HuD^+^ cells was significantly increased in VPA-raised tadpoles compared to Ctrl. (Ctrl, 4379.8 ± 134.8, *N* = 5; VPA, 4791.8 ± 89.7, *N* = 5; *p < 0.05). **(D)** Representative images of apoptosis in control and VPA-reared optic tecta. Arrows indicate the apoptotic cells. Scale bar = 50 μm. **(E)** The data shows that the number of apoptotic cells was significantly decreased in VPA-raised tadpoles compared to Ctrl. (Ctrl, 62.2 ± 3.5, *N* = 6; VPA, 30.6 ± 5.5, *N* = 5; ***p < 0.001). **(F)** Control and VPA-reared optic tecta were performed TUNEL analysis and immunostained with an anti-GABA antibody at stage 48. Arrows indicate that the apoptotic cells are not stained with GABA. (Ctrl, *N* = 6; VPA, *N* = 7). Scale bar = 50 μm. *N* represents the number of tadpoles.

The optic tectum is mainly assembled by differentiated neurons and progenitor cells (Sharma and Cline, 2010; Tao et al., 2015; Herrgen and Akerman, 2016). To determine whether GABA^+^ cells are differentiated neurons, we used a pan-neuronal marker HuD to characterize the cell identities (Figure 4A). We found that GABA^+^ cells were HuD^+^ neurons in control (Figures 4A1–A4) and VPA-treated (Figures 4A5–A8) optic tectum. VPA-induced increases in GABA^+^ cells (Figure 4B) were also HuD^+^ neurons (Figure 4C).

To test whether VPA treatment affects cell survival or not, we exposed tadpoles at stage 46 to VPA for 48 h and fixed them for TUNEL analysis. The total apoptotic cells in the optic tectum were measured within 5 representative brain slices (see experimental procedures for details). We found that the majority of apoptotic cells were distributed in the cell body layer of the tectum (Figure 4D). Interestingly, VPA treatment greatly decreased the number of apoptotic cells (Figure 4E), suggesting that VPA (1 mM) incubation for 48 h in our experimental conditions may exert a neuroprotective effect on the tectal cells. We counterstained the slices with an anti-GABA antibody after TUNEL staining to determine whether the apoptotic cells were GABAergic cells. We did not find any significant co-staining of GABA with TUNEL analysis both in Ctrl and VPA-treated tadpoles (Figure 4F), suggesting that the VPA-induced increase of GABAergic neurons is not due to a decrease of apoptotic cells.

### Valproate acid increases GABAergic neurotransmission

**FIGURE 5:**
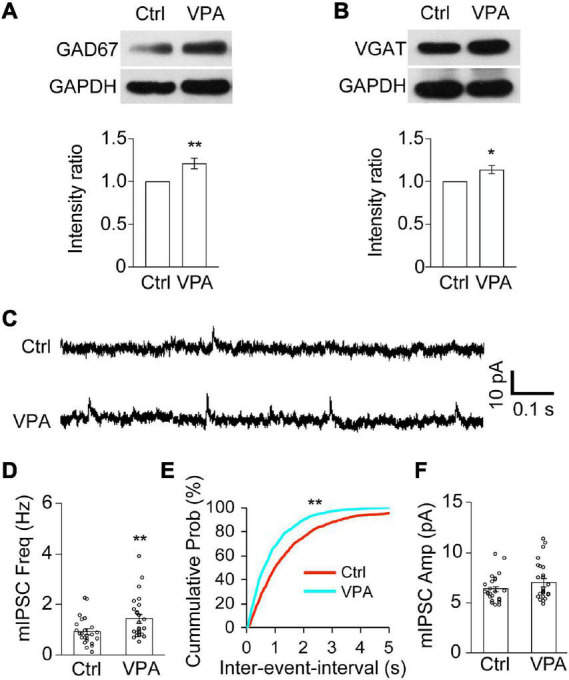
Valproate acid increases GABAergic synapses and the frequency of mIPSCs. **(A,B)** Western blot analysis and data summary show that the relative intensities of GAD67 **(A)** and VGAT **(B)** to GAPDH were significantly increased in VPA-reared tadpoles compared to Ctrl. GAD67, *N* = 6; VGAT, *N* = 6. *N* represents the number of independent experiments. *p < 0.05, **p < 0.01. **(C)** Representative traces were showing the recordings of mIPSCs from Ctrl and VPA-treated animals. Scale bar = 10 pA, 0.1 s. **(D–F)** The data shows that the frequency of mIPSCs was significantly increased in VPA-reared tadpoles compared to control tadpoles **(D)**. **p < 0.01. The cumulative probability plot shows that the inter-event-interval of mIPSCs was significantly decreased in VPA-treated tadpoles compared to ctrl **(E)**. Kolmogorov–Smirnov test, **p < 0.01. The data shows that the amplitude of mIPSCs was not altered in VPA-reared tadpoles compared to control tadpoles **(F)**. Ctrl, *N* = 24, *n* = 5; VPA, *N* = 24, *n* = 4. *N* represents the number of neurons and *n* represents the number of tadpoles.

To investigate whether VPA changes GABAergic neurotransmission, we measured the protein expression of GABA synthesizing enzyme, 67 kDa isoform of glutamic acid decarboxylase (GAD67) (Figure 5A). We found that the protein level of GAD67 was significantly increased in the VPA-treated tadpoles compared to control tadpoles (Figure 5A). The protein expression of vesicular GABA transporter (VGAT), a well-established inhibitory presynaptic terminal marker, was also significantly increased in VPA-reared animals compared to control animals (Figure 5B). Combined with the changes in GAD67 upon VPA treatment, it suggested that the GABA synthesis and transport might be enhanced upon VPA treatment.

To functionally determine whether epigenetic regulation alters GABAergic synaptic transmission in the developing brain, we recorded GABA_*A*_R-mediated miniature inhibitory postsynaptic currents (mIPSCs) in the VPA-reared tadpoles at stage 46 for 48 h by holding membrane potential at 0 mV (Figure 5C). We found that the frequency of mIPSCs was significantly greater in the VPA-raised tadpoles compared to the control tadpoles (Figures 5D,E). In contrast, the amplitudes of mIPSCs were not statistically different (Figure 5F). These results suggested that VPA may increase GABAergic synaptic neurotransmission in the developing brain.

### Valproate acid interferes with swimming and avoidance behavior

**FIGURE 6:**
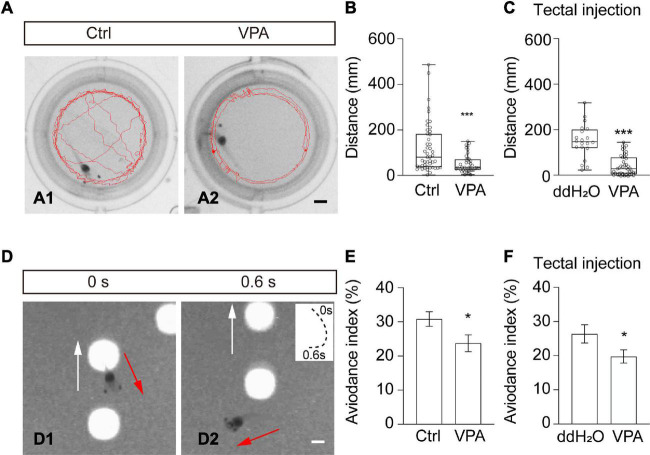
Valproate acid-reared tadpoles show impaired free-swimming and visual avoidance behaviors. **(A)** Representative images showing the swimming traces of a tadpole within 60 s in Ctrl (left) and VPA-reared (right) tadpoles. Scale bar = 2 mm. **(B)** Summary of data showing the total swimming distance was decreased in VPA-raised tadpoles compared to ctrl tadpoles. (Ctrl, 120.3 ± 16.3 mm, *N* = 47; VPA, 51.1 ± 6.3 mm, *N* = 41; ***p < 0.001). **(C)** The data summary shows that the total swimming distance decreased in VPA-injected tadpoles compared to ddH_2_O-injected tadpoles. (ddH_2_O, 187.4 ± 16.9 mm, *N* = 20; VPA, 78.5 ± 6.5 mm, *N* = 45; ***p < 0.001). **(D)** A sequence of time-lapse images shows that a tadpole makes an avoiding turning in response to a moving spot (4 mm diameter) within 0.6 s. White arrows indicate the moving direction of the spot. Red arrows indicate the turning direction of the tadpole. The panel on the upper right shows the swim trajectories of the animal, with the start point marked as 0 s and the end point marked as 0.6 s. Spot luminance: 90 cd/m^2^. Scale bar = 2 mm. **(E)** Summary of data showing that the avoidance index (AI,%) was significantly decreased in VPA-treated tadpoles compared to Ctrl tadpoles. (Ctrl, 30.8 ± 2.2, *N* = 24; VPA, 23.8 ± 2.5, *N* = 24; *p < 0.05). **(F)** The data shows that the avoidance index was significantly decreased in VPA-injected tadpoles compared to ddH_2_O-injected tadpoles. (ddH_2_O, 28.5 ± 2.8, *N* = 20; VPA, 21.1 ± 2.0, *N* = 36; *p < 0.05). *N* represents the number of tadpoles.

The optic tectum is the processing center for visual information and swimming behavior (Dong et al., 2009; Shen et al., 2011). To test the functional effects of VPA on tadpole behavior, we first measured the swimming behavior of tadpoles in a behavioral setup (Figure 6A). In Steinberg’s control medium-reared group, the tadpoles tended to swim randomly in the recording chamber (Figure 6A1). In VPA-reared tadpoles, animals were likely to swim along the wall of the chamber (Figure 6A2). The swimming speed (Ctrl: 2.0 ± 0.3 mm/s, *N* = 47; VPA: 0.9 ± 0.1 mm/s; *N* = 41; ^***^p < 0.001) and total swim distance (Figure 6B) in VPA-treated tadpoles were significantly decreased compared to control tadpoles within 60 s, indicating that VPA-reared tadpoles were less active than control tadpoles. To specifically examine the effects of VPA on the optic tectum, we injected VPA (1 mM) into the tectal ventricle and performed a behavioral test after 48 h. We found that the swimming speed (Tectal injection: ddH_2_O, 3.1 ± 0.3 mm/s, *N* = 20; VPA, 1.3 ± 0.1 mm/s, *N* = 45; ^***^p < 0.001) and total swim distance (Figure 6C) in VPA-injected tadpoles were significantly decreased compared to ddH_2_O-injected control tadpoles, confirming that VPA affects swimming behavior by interfering neural circuit.

To determine whether VPA treatment would also affect visually guided avoidance behavior, we incubated or injected tadpoles with VPA for 48 h. The avoidance index (AI) was counted as the percentage of tadpoles that avoided the first ten approaching moving spots (4 mm diameter) (Figure 6D). The AI values were greatly decreased both in VPA-reared (Figure 6E) and VPA-injected (Figure 6F) tadpoles compared to control tadpoles. These results showed that VPA blocked swimming activity and visually guided avoidance behavior.

## Discussion

In this study, we provide evidence that GABAergic neurons developmentally decrease in the later developing vertebrate brain. The acetylation of H3K9 also decreases between the stages from 46 to 48. VPA treatment significantly increases the H3K9 acetylation level and the number of GABAergic neurons without inducing apoptosis. Importantly, we provide electrophysiological evidence showing that the frequency of mIPSCs is increased in the VPA-treated *Xenopus* brain. Moreover, VPA treatment interrupts the animal’s swimming and avoidance behavior. Our *in vivo* findings underscore the importance of epigenetic control of GABAergic differentiation, neurotransmission, and behavior during early brain development.

GABAergic cells constitute about 10–20% of all neurons in the cortex and vary from 3 to 60% regarding differential spatial populations (Caputi et al., 2013). We found that about 30% of tectal cells are GABA immunoreactive at stages 42–46, consistent with previous reports (Miraucourt et al., 2012). It is well known that GABAergic neurons develop earlier than glutamatergic neurons (Ben-Ari, 2002; Deng et al., 2007) and induce depolarization of progenitor cells, which facilitate NMDA receptors to promote neural differentiation and brain maturation (Ben-Ari, 2002; Akerman and Cline, 2006; Ge et al., 2007). GABAergic neurons become more mature and efficient with age to sharpen the spatiotemporal precision of spiking, as revealed by the developmental decrease of the inhibitory receptive field and instructive effects of GABAergic transmission (Tao and Poo, 2005; Richards et al., 2010; Shen et al., 2011). The decrease of GABAergic cells from 30 to 13% during early development may partly be attributable to the refinement of neural circuits (Tao and Poo, 2005; Akerman and Cline, 2006).

Emerging evidence demonstrates that various factors control the GABAergic cell fate before and during the embryonic stages of development (Batista-Brito et al., 2008). VPA, a broad-spectrum HDAC inhibitor, has been demonstrated to cause hyperacetylation and increase the differentiation of cells in several cell cultures or animal models (Hsieh et al., 2004). VPA can potentiate GABAergic activity at synapses by inhibiting GABA transaminase, an enzyme accounted for the degradation of GABA. GABA synthesizing enzyme glutamic acid decarboxylase (GAD67) and vesicular GABA transporter (VGAT) are responsible for GABA synthesis and its transport into synaptic vesicles. GABA_*A*_ receptor subunits, GAD65/67 and KCC2, are key factors that affect the development of GABAergic inhibitory neurons. Since GAD67 is a rate-limiting enzyme responsible 90% of GABA synthesis in neurons, alterations of GAD67 will affect the cellular and vesicular GABA content (Huang, 2009; Miraucourt et al., 2012). We found that GAD67 and VGAT protein expressions are greatly increased by VPA treatment. VPA exposure also increases the number of GABAergic neurons and the frequency of mIPSCs, suggesting that VPA can increase GABAergic synaptic inhibition (Löscher, 1999). Given that VPA treatment decreases the number of BrdU-positive progenitor cells in the optic tectum (Tao et al., 2015), it is therefore conceivable that VPA plays dual effects on GABAergic differentiation at the expense of progenitor cells (Laeng et al., 2004). However, we could not exclude the possibility that VPA treatment could switch the cell fate from glutamatergic to GABAergic neurons, which needs to be further determined in future studies.

Coordinated development of excitatory and inhibitory inputs is required to establish a functional neural circuit, as revealed by the developmental refinement of receptive fields (Tao and Poo, 2005; Shen et al., 2011). Balanced inhibition serves as an essential regulator to increase temporal precision and decrease random current noise (Wehr and Zador, 2003; Shen et al., 2011). Selective blockade of inhibitory inputs using a γ2 subunit intracellular loop disrupts the balance of excitation to inhibition and visual avoidance behavior, accompanied by the loss of temporal fidelity in the tectal neurons (Shen et al., 2009, 2011). Our electrophysiological recordings show that VPA treatment induces an increase in the frequency of mIPSCs, suggesting that upregulation of histone acetylation not only increases the number of GABAergic neurons but also enhances the GABAergic synaptic transmission. The optic tectum is a visual processing center that instructs visually guided behavior (Dong et al., 2009; Khakhalin et al., 2014; Shen et al., 2014). Dysfunction of GABAergic neurotransmission affects the early formation of neural circuits and experience-dependent neural plasticity (Huang, 2009; Shen et al., 2009; Ramamoorthi and Lin, 2011). It is reasonable to conclude that a VPA-induced increase of GABAergic transmission in the *Xenopus* may interfere with the visual information processing, resulting in disrupted swimming behavior (James et al., 2015). As VPA has profound effects on excitatory and inhibitory neurotransmission, the disruption of the balance between neuronal excitation and inhibition could be the potential cause of behavioral deficits (Eichler and Meier, 2008; Shen et al., 2011; Zhang and Sun, 2011).

Epigenetic regulation has been established to correlate with transcriptional activation or repression through histone acetylation or deacetylation. Class I HDACs have divergent actions on cell proliferation, differentiation, and neural development (Gao et al., 2016b; Ruan et al., 2017). HDAC1 has been shown to regulate GABAergic synaptic transmission (Ruan et al., 2017). Combined with the data showing that VPA increases GABAergic synaptic transmission and promotes the maturation of GABAergic neurons, we propose that VPA directly acts on HDACs to induce changes in transcription machinery, resulting in enhanced GABAergic neurogenesis-related gene expressions. It is intriguing to study further the specific HDACs involved in regulating GABAergic neurogenesis and behavioral deficits. Thus, it seems plausible that VPA induces activation of GAD67 and VGAT, increasing the number of GABAergic synapses and enhancing the GABAergic inhibitory neurotransmission. However, what gene expressions are involved in the VPA-induced increase of GABAergic neurons and neurotransmission is still unclear. Previous studies used RNA sequencing to screen up- and down-regulated genes in the VPA-treated cells (Fukuchi et al., 2009). Uncovering the signaling mediated by histone acetylation would help to decipher the epigenetic mechanism that regulates GABAergic cell development.

## Data availability statement

The raw data supporting the conclusions of this article will be made available by the authors, without undue reservation.

## Ethics statement

The animal study was reviewed and approved by the local Ethics Committee of the Hangzhou Normal University. Written informed consent was obtained from the owners for the participation of their animals in this study.

## Author contributions

JG, MQ, and WS: study concept and design. JG, YHL, YFL, and WS: acquisition of data. JG, YHL, and WS: analysis and interpretation of data. JG and WS: statistical analysis. WS: drafting of the manuscript. All authors had full access to all the data in the study and took responsibility for the integrity of the data and the accuracy of the data analysis.
